# Electroacupuncture prevents cocaine-induced conditioned place preference reinstatement and attenuates ΔFosB and GluR2 expression

**DOI:** 10.1038/s41598-021-93014-0

**Published:** 2021-07-01

**Authors:** Ai T. M. Nguyen, Tran V. B. Quach, Peddanna Kotha, Szu-Yu Chien, Iona J. MacDonald, Hsien-Yuan Lane, Cheng-Hao Tu, Jaung-Geng Lin, Yi-Hung Chen

**Affiliations:** 1 School of Chinese Medicine, College of Chinese Medicine, China Medical University, Taichung, Taiwan; 2 Graduate Institute of Acupuncture Science, College of Chinese Medicine, China Medical University, Taichung, Taiwan; 3 Graduate Institute of Biomedical Sciences, College of Medicine, China Medical University, Taichung, Taiwan; 4 Department of Psychiatry, China Medical University Hospital, Taichung, Taiwan; 5 Chinese Medicine Research Center, China Medical University, Taichung, Taiwan; 6 Department of Psychology, College of Medical and Health Science, Asia University, Taichung, Taiwan; 7 Department of Photonics and Communication Engineering, Asia University, Taichung, Taiwan

**Keywords:** Health care, Medical research, Neurology

## Abstract

Acupuncture has been used for treating drug addiction since the 1970s, but little is known about the mechanisms by which acupuncture affects drug cue-induced relapse. The transcription factor delta-FosB (ΔFosB) plays a critical role in behavior and pathology after chronic use of cocaine. ΔFosB regulates glutamate receptor signaling and dendritic spine morphology in animal models. This experimental study compared the effects of electroacupuncture (EA) at acupoints LI4 and LI11 with those of another potentially beneficial intervention, gabapentin (GBP), alone or in combination, on reinstatement of cocaine-induced conditioned place preference (CPP) and levels of ΔFosB and glutamate receptor subunit 2 (GluR2) expression in the nucleus accumbens (NAc). EA at LI4 and LI11 significantly prevented cue-induced cocaine CPP reinstatement, whereas needle insertion without electrical stimulation at these acupoints had no such effect. EA also significantly attenuated cocaine-induced increases in ΔFosB and GluR2 expression in the NAc. Unexpectedly, these effects were reversed when GBP was combined with EA. Treatment with EA at LI4 and LI11 prevented cocaine-induced increases in dendritic spine density in the NAc core and shell. Our results suggest that EA at LI4 and LI11 may prevent cocaine relapse by modulating ΔFosB and GluR2 expression, as well as dendritic spine density.

## Introduction

Drug abuse is a huge problem for communities and health care systems worldwide^1^. Chronic abuse of addictive substances leads to neuroplasticity or neuroadaptation in brain structure and function, impairing cognitive functions, making it difficult for individuals to stop using the addictive substances and rendering them highly susceptible to relapse, even after long periods of abstinence^1,2^. More than two-thirds of people diagnosed with substance use disorder relapse within the first year after undergoing detoxification^1,3,4^. Cocaine is a highly addictive psychostimulant and no regulatory authorities worldwide have as yet approved any medications for the treatment of addiction to cocaine in adolescents and adults. Cocaine’s rewarding effects are mediated by activation of the mesocorticolimbic dopamine system^5^, consisting of the ventral tegmental area (VTA), the nucleus accumbens (NAc)^6^, prefrontal cortex (PFC) and amygdala^7^. The NAc has a critical role in the reward circuitry underlying addiction, as it receives dopaminergic input from the VTA and glutamatergic input from the hippocampus, amygdala and frontal cortex^8^. Previous studies have demonstrated that the transcription factor delta-FosB (ΔFosB) plays a critical role in behavior and pathology after chronic use of cocaine^9–11^. ΔFosB regulates the glutamate receptor and the morphology of dendritic spine and neural plasticity in animal models of addiction^9,12,13^. Acute cocaine administration increases c-Fos expression in the NAc, while chronic cocaine use increases ΔFosB expression and changes behavioral plasticity by altering the expression of its target genes: alpha-amino-3-hydroxy-5-methyl-4-isoxazolepropionic acid (AMPA) glutamate receptor subunit 2 (GluR2), cyclin-dependent-kinase 5 (Cdk5) and the opioid peptide, dynorphin^14,15^. The overexpression of ΔFosB protein induced by chronic cocaine administration leads to changes in the glutamatergic synapses in the NAc, including increased dendritic spine density^9^. ΔFosB is believed to be capable of converting acute drug-induced responses over time into relatively stable adaptations that contribute to long-term neural and behavioral plasticity underlying addictive behavior^16^. Whether any correlation exists between the overexpression of GluR2 and ΔFosB in priming-induced reinstatement of cocaine-seeking behavior in animals has yet to be determined.

Acupuncture needle penetration into acupuncture points (or acupoints) at specific locations of the body is followed by either manual manipulation (traditional acupuncture) or electrical current stimulation (electroacupuncture [EA]). Acupuncture has been used to treat opiate addiction since the 1970s, although its efficacy in drug addiction treatment needs to be confirmed by large-scale clinical trials^17–19^. Moreover, the mechanisms of these different acupuncture modalities that are used in cocaine addiction treatment have not been clarified. Acupuncture at the HT7 (Shenmen) acupoint in rats decreases the release of dopamine, enhances gamma-aminobutyric acid-ergic (GABAergic) inhibition and suppresses morphine- and cocaine-induced c-Fos overexpression^20^, while acupuncture at ST36 (Zusanli) attenuates alcohol-induced increases in FosB/ΔFosB immunoreactivity within the rat dorsolateral striatum and NAc core^21^. Furthermore, animal studies have shown that EA reduces the risk of relapse to drug-seeking behavior. Thus, the experimental evidence shows that acupuncture modulates molecular abnormalities induced by exposure to addictive substances, but evidence is lacking as to the effects of acupuncture on glutamatergic neurotransmission and how acupuncture reduces the reinstatement of drug-seeking behavior. The lack of standardized therapeutic protocols for acupuncture treatment of drug addiction complicates comparisons of outcomes from existing acupuncture studies using different formulas^17^.

Clinical evidence suggests that the anticonvulsant gabapentin (GBP) may be helpful in treating alcohol dependence and for reducing the symptoms of insomnia, dysphoria and craving^22–24^, in opioid addiction^25–27^, and in treating cannabis dependence^28^. One small, open-label trial has described finding that GBP reduced cocaine use and craving^29^, although other research has reported that GBP does not affect abstinence rates, treatment retention, cravings, the subjective effects of cocaine, or likelihood of future cocaine use^30–34^. Interestingly, the combination of GBP and EA at ST36 was associated with more robust analgesia than either GBP or EA alone in mice subjected to paclitaxel-induced neuropathic pain^35^. We therefore sought to determine whether treatment with combined GBP and EA would have any beneficial effect upon cue-induced cocaine seeking and relapse.

Some researchers have suggested that some of the persistent neurobehavioral consequences of repeated exposure to psychostimulant drugs may be because these agents are capable of reorganizing synaptic connectivity patterns in the NAc and PFC^12^. For instance, cocaine doubles the numbers of branched spines and increases dendritic spine density on medium spiny neurons in rats^12^. To the best of our knowledge, no studies have reported the effects of acupuncture on dendritic spine morphology in drug addiction. We speculated that exploring the effects of acupuncture treatment on dendritic spine morphology may lead to more effective treatment of drug addiction disorders.

In the present study, we compared the effects of EA at acupoints LI4 (Hegu) and LI11 (Quchi) with those of GBP, alone or in combination, on cocaine-induced conditioned place preference (CPP) reinstatement, ΔFosB and GluR2 expression, as well as dendritic spine density in the mouse NAc.

## Results

### Only EA at LI4 and LI11 prevents cocaine-induced CPP reinstatement

**Figure 1:**
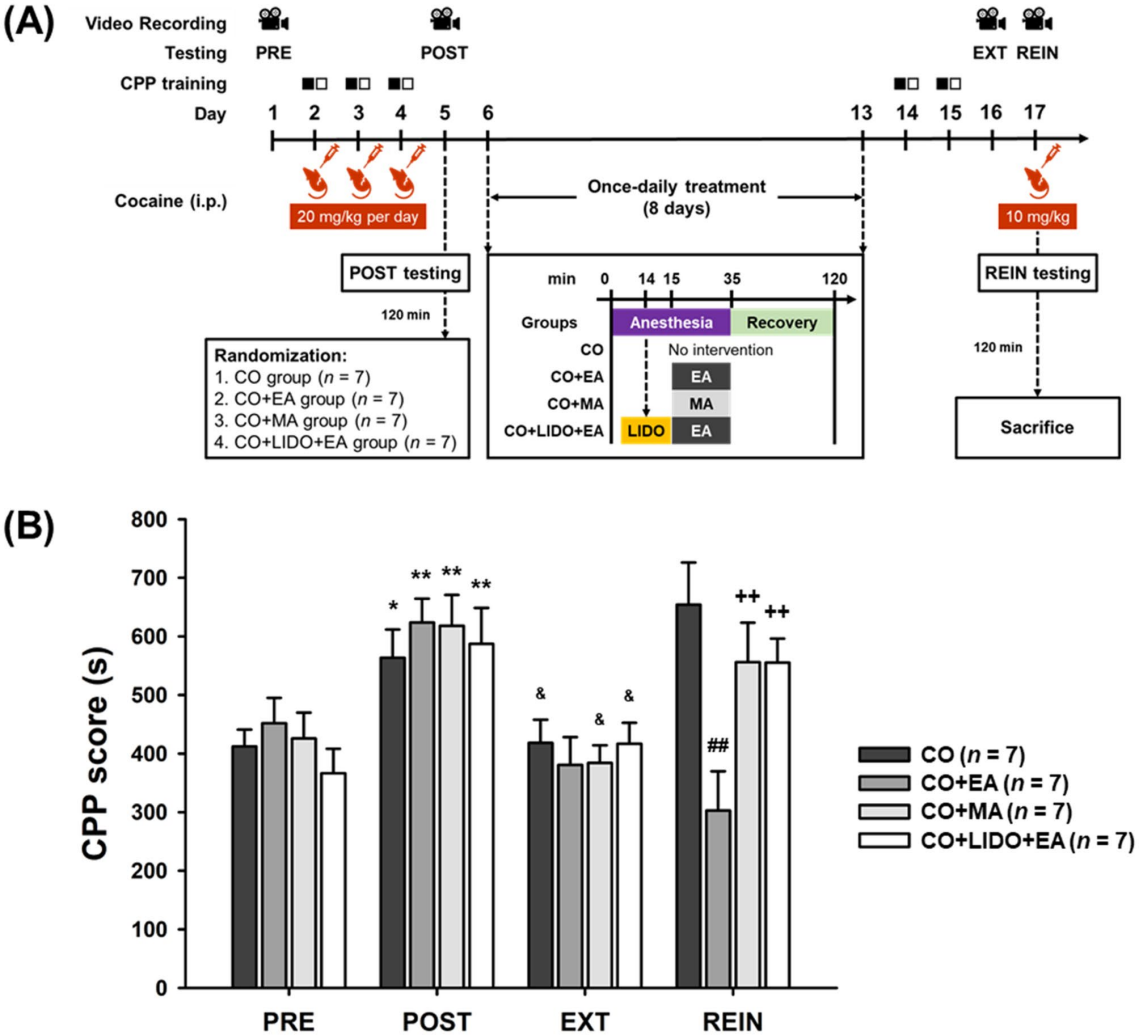
EA at LI4 and LI11 acupoints prevents cocaine-induced CPP reinstatement in mice. (**A**) Timeline for the CPP testing, training, cocaine administration, and experimental interventions. (**B**) CPP scores for the CO group, CO + EA group, CO + MA group, and CO + LIDO + EA group, obtained during PRE, POST, EXT, and REIN testing. All data are expressed as the means ± S.E.M. (*n* = 7 mice per group). *p < 0.05; **p < 0.01 versus the PRE phases; ^&^*p* < 0.05 versus the REIN phases; ^##^*p* < 0.01 versus the CO group; ^++^*p* < 0.01 versus the EA group (two-way ANOVA with the LSD post-hoc test). Abbreviations: CPP = Cocaine-induced conditioned place preference; PRE = Preconditioning testing; POST = Post-conditioning testing; EXT = Extinction of cocaine CPP testing; REIN = Cocaine-primed reinstatement testing; CO = No intervention; CO + EA = EA treatment; CO + MA = MA treatment; CO + LIDO + EA = Lidocaine (2%, 10 μL) injection followed by EA treatment.

The cocaine doses of 20 mg/kg for conditioning and 10 mg/kg for reinstatement followed those described in previous reports^36–39^. In CPP testing, approximately 90% of the mice preferred the black chamber on the pre-testing day; these mice were included in the study, while the remaining animals preferred the other side and were excluded from the study. The timeline for the experiment is shown in Fig. 1A. Two-way analysis of variance (ANOVA) revealed significant interaction effects (*F*[9,72] = 4.031, *p* < 0.001) between groups (no intervention [CO], EA treatment [CO + EA], manual acupuncture (MA) treatment [CO + MA], and 2% lidocaine injection followed by EA treatment [CO + LIDO + EA]) and phases (preconditioning testing [PRE], post-conditioning testing [POST], extinction testing [EXT], and reinstatement testing [REIN]). In group factor analysis, significant between-group differences were observed with the REIN phase only (*F*[3,24] = 5.690, *p* < 0.01). No significant between-group differences were observed with the PRE, POST, or EXT phases. Post-hoc analysis of the REIN phase revealed significantly decreased CPP scores in the CO + EA group, compared with the CO group (*p* < 0.01), the CO + MA group (*p* < 0.05) and the CO + LIDO + EA group (*p* < 0.05), respectively. No significant differences were seen with any other pairs of treatment groups. In phase factor analysis, significant differences between phases were seen for all four groups (CO group: *F*[3,18] = 5.298, *p* < 0.01; CO + EA group: *F*[3,18] = 12.754, *p* < 0.001; CO + MA group: *F*[3,18] = 7.963, *p* < 0.01; CO + LIDO + EA group: *F*[3,18] = 7.969, *p* < 0.01). Post-hoc analysis revealed that CPP scores were significantly increased in the POST phase compared with the PRE phase in all groups (CO group: *p* < 0.05; CO + EA group: *p* < 0.01; CO + MA group: *p* < 0.01; CO + LIDO + EA group: *p* < 0.01), whereas CPP scores were significantly decreased in the EXT phase compared with the POST phase (CO group: *p* < 0.05; CO + EA group: *p* < 0.001; CO + MA group: *p* < 0.01; CO + LIDO + EA group: *p* < 0.05). No significant difference was found between the PRE phase and EXT phase. In contrast, the CPP score was significantly increased in the REIN phase compared with the EXT phase in the CO group, the CO + MA group, and in the CO + LIDO + EA group (all *p* < 0.05), but not in the CO + EA group. Furthermore, CPP scores were significantly decreased in the REIN phase compared with the POST phase in the CO + EA group (*p* < 0.01), but not in the CO group, the CO + MA group, and in the CO + LIDO + EA group. These results suggest that EA at LI4 and LI11, but not manual needling at LI4 and LI11, prevents cocaine-primed CPP reinstatement. The effects of EA at LI4 and LI11 were significantly blocked by pretreatment with lidocaine (2%) at LI11 given 1 min before EA treatment (*p* < 0.05; Fig. 1B and Supplementary Fig. S1).

### Gabapentin reversed EA-induced effects in cocaine-induced CPP reinstatement

**Figure 2:**
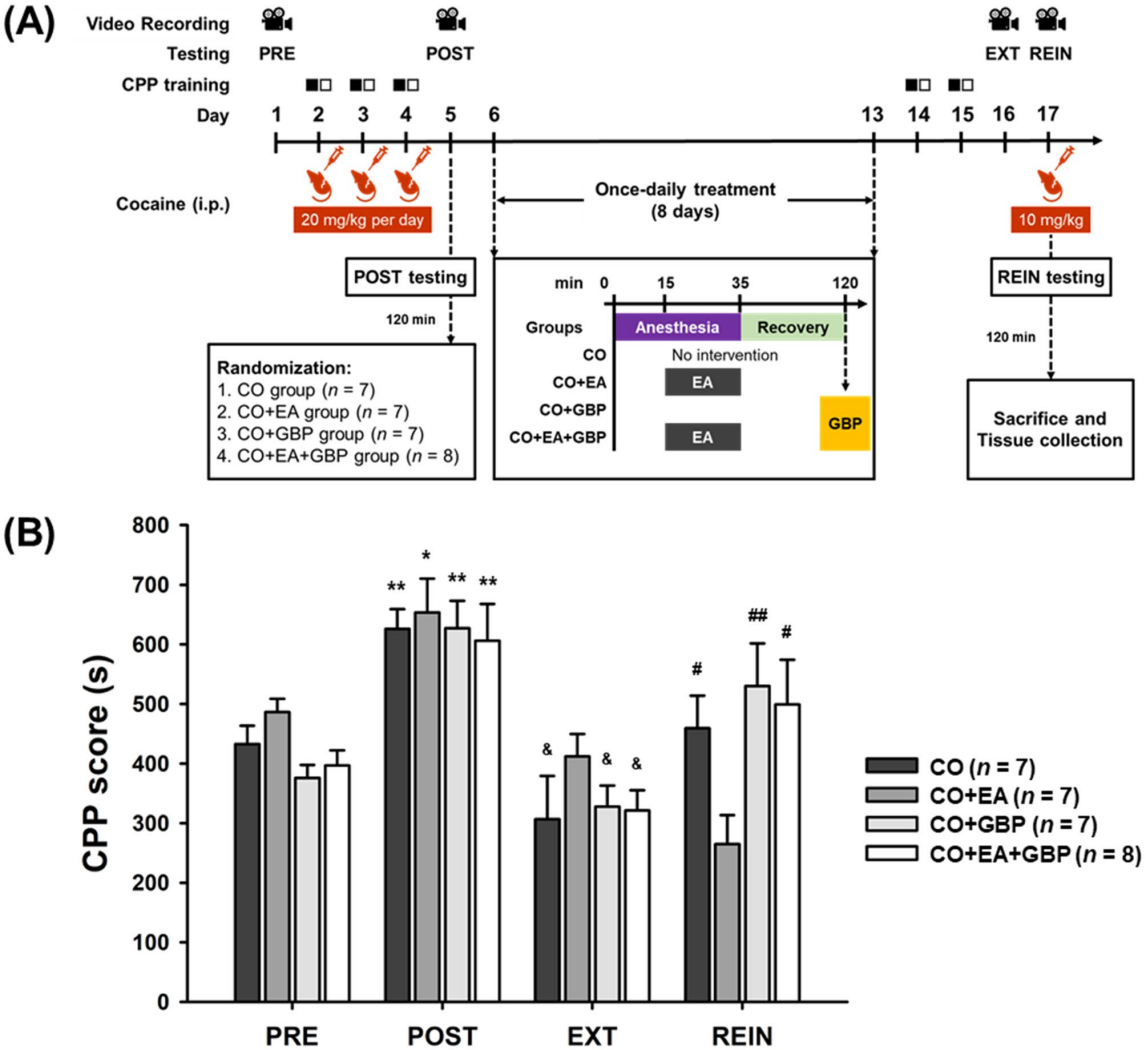
GBP significantly reversed the effects of EA on cocaine-induced relapse behavior. (**A**) Timeline of CPP testing, training, cocaine administration, and experimental interventions. (**B**) CPP scores for the CO group, CO + EA group, CO + GBP group and CO + EA + GBP group, obtained during PRE, POST, EXT, and REIN testing. *p < 0.05; **p < 0.01 versus the PRE phases; ^&^*p* < 0.05 versus the REIN phases; ^#^*p* < 0.05; ^##^*p* < 0.01 versus the CO + EA group in REIN phases (two-way ANOVA with the LSD post-hoc test). All data are expressed as the means ± S.E.M. (*n* = 7–8 mice per group). Abbreviations: CPP = Cocaine-induced conditioned place preference; PRE = Preconditioning testing; POST = Post-conditioning testing; EXT = Extinction of cocaine CPP testing; REIN = Cocaine-primed reinstatement testing; CO = No intervention; CO + EA = EA treatment; CO + GBP = i.p. GBP (1 mg/kg) injection; CO + EA + GBP = EA treatment followed by i.p. GBP (1 mg/kg) injection.

In a previous study, GBP (1–30 mg/kg) attenuated hyperactivation induced by cocaine (10 mg/kg) in rats^40^. We therefore tested the effects of GBP at the dose of 1 mg/kg (see Fig. 2A for the timeline). We speculated that the combination of GBP plus EA at LI4 and LI11 would enhance the effects of EA on the reduction of CPP in cocaine-treated mice in the REIN stage. Two-way ANOVA revealed significant interaction effects between groups (no intervention [CO], EA treatment [CO + EA], intraperitoneal (i.p.) 1 mg/kg GBP treatment [CO + GBP], and EA followed by i.p. GBP [CO + EA + GBP]) and phases (PRE, POST, EXT, and REIN) (*F*[6.354, 52.948] = 2.954 with Greenhouse–Geisser correction for sphericity, *p* < 0.05). For group factor analysis, the REIN phase revealed significant differences between groups (REIN phase: *F*[3,25] = 3.344, *p* < 0.05). In the REIN phase, CPP scores were significantly lower in the CO + EA group compared with those in the CO group (*p* < 0.05), the CO + GBP group (*p* < 0.01) and the CO + EA + GBP group (*p* < 0.05). Factor analysis revealed significant between-phase differences in all groups (CO group: *F*[1.443, 8.656] = 8.085 with Greenhouse–Geisser correction for sphericity, *p* < 0.05; CO + EA group: *F*[3,18]= 14.674, *p* < 0.001; CO + GBP group: *F*[3,18] = 11.637, *p* < 0.001; CO + EA + GBP group: *F*[3,21] = 5.785, *p* < 0.01).

Post-hoc analysis revealed significant increases in CPP scores in the POST phase compared with the PRE phase (CO group: *p* < 0.01; CO + EA group: *p* < 0.05; CO + GBP group: *p* < 0.01; CO + EA + GBP group: *p* < 0.01) and the EXT phase (CO group: *p* < 0.05; CO + EA group: *p* < 0.01; CO + GBP group: *p* < 0.01; CO + EA + GBP group: *p* < 0.01) for all groups. Moreover, CPP scores were significantly higher in the REIN phase compared with the EXT phase in the CO group, the CO + GBP group, and the CO + EA + GBP group (all *p* < 0.05), but not the CO + EA group. Thus, while EA at LI4 and LI11 significantly reduced the CPP score, adding GBP to EA significantly reversed the effects of EA (Fig. 2B and Supplementary Fig. S2).

### Effects of EA at LI4 and LI11 on ΔFosB and AMPA GluR2 protein expression in the NAc

**Figure 3:**
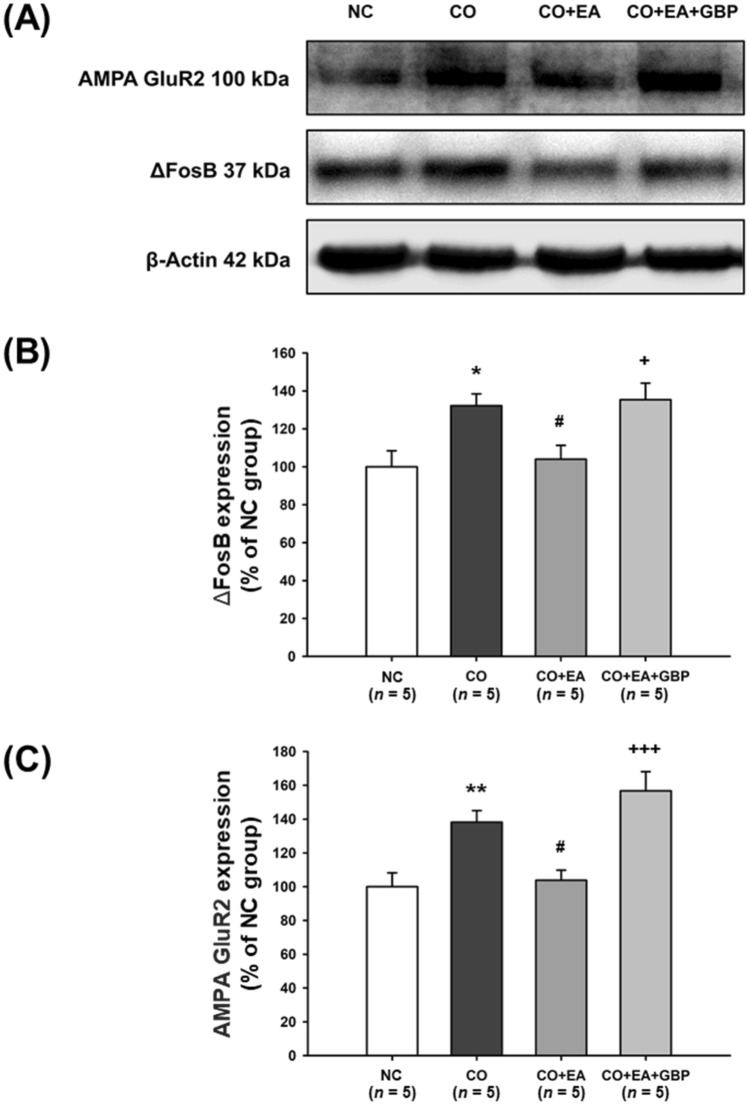
EA treatment attenuated cocaine-induced increases of ΔFosB and GluR2 levels in the NAc. After 2 h of REIN testing, the mice were sacrificed and NAc tissue was collected from the brain, for quantification of protein levels in the NAc. Figure 2A shows the experimental timeline. (**A**) Following the study group interventions, Western blotting examined ΔFosB and GluR2 expression. The NC group (without any treatment) was used to confirm the basal levels of protein. The original Western blot images are available in Supplementary Figure S10, with photographs of each membrane taken after cropping. Data were calculated as the percentages of the NC group and are expressed as the means ± S.E.M. (*n* = 5 mice per group). (**B**) Quantification of ΔFosB expression from (**A**). *p < 0.05 versus NC group; ^#^*p* < 0.05 versus the CO group; ^+^*p* < 0.05 versus the CO + EA group (one-way ANOVA with the LSD post-hoc test). (C) Quantification of GluR2 expression from Fig. 3A. **p < 0.01 versus NC group; ^#^*p* < 0.05 versus the CO group; ^+++^*p* < 0.001 versus the CO + EA group (one-way ANOVA with the LSD post-hoc test). Abbreviations: NC = Negative control; CO = No intervention; CO + EA = EA treatment; CO + EA + GBP = EA treatment followed by i.p. GBP (1 mg/kg) injection.

**Figure 4:**
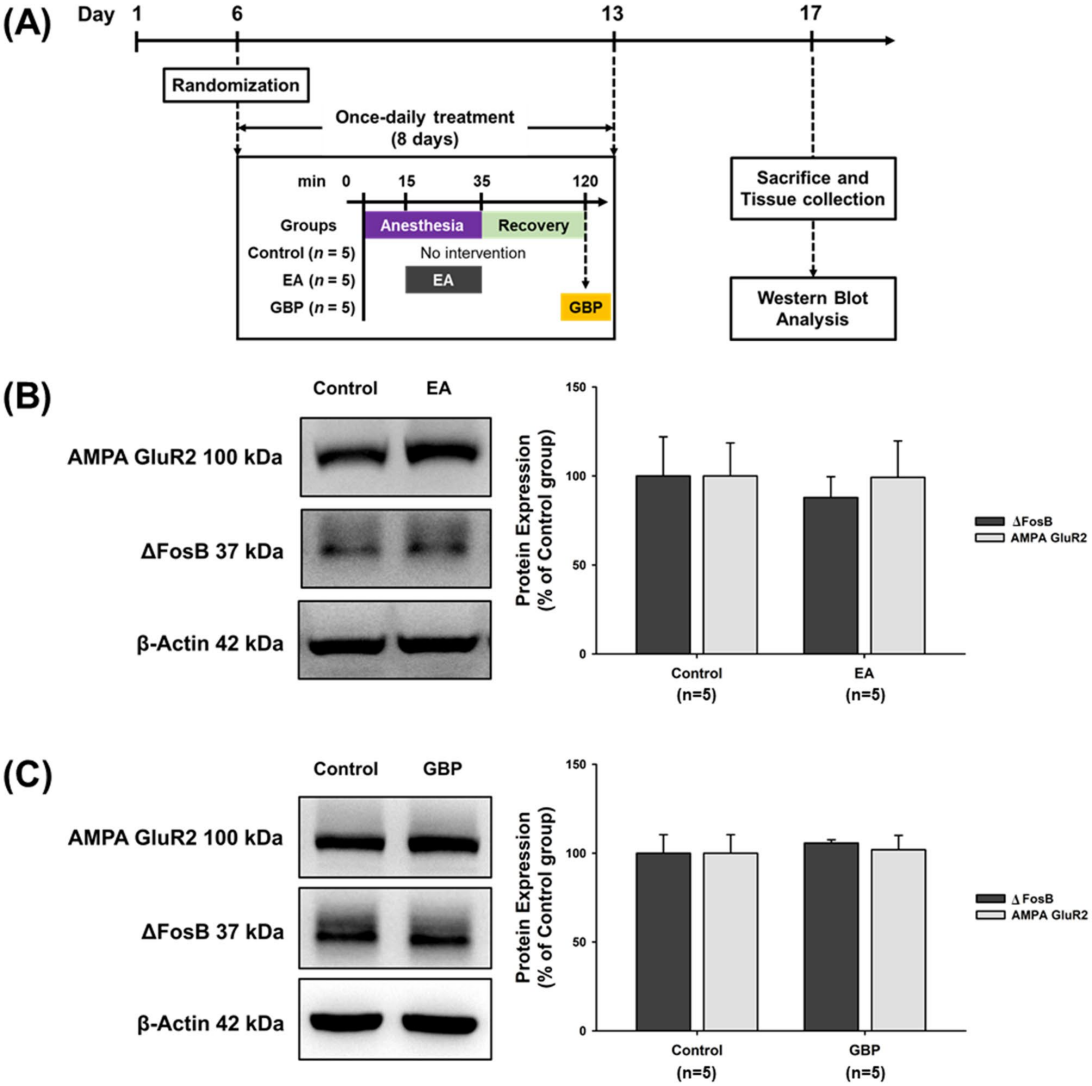
EA or GBP alone did not affect ΔFosB and GluR2 expression in cocaine-naïve mice. (**A**) Timeline for the experimental procedures. (**B**, **C**) Cocaine-naïve mice were treated with EA or GBP (1 mg/kg i.p.). Levels of ΔFosB and GluR2 expression were examined by Western blotting (*n* = 5 per group). The original Western blot images are available in Supplementary Figure S11 and S12, respectively. The bar graphs show the quantification of protein from (**B**, **C**). The same control group was used for both Western blotting assays. Abbreviations: Control = Cocaine-naïve mice; EA = cocaine-naïve mice with EA treatment; GBP = Cocaine-naïve mice with i.p. GBP (1 mg/kg) injection.

In Fig. 3A, one-way ANOVA revealed a significant main effect for the group factor in the Western blot analysis for ΔFosB (*F*[3,16] = 5.693, *p* < 0.01) and GluR2 (*F*[3,16] = 10.829, *p* < 0.001) expression (Fig. 3B, C; Supplementary Fig. S3 and S4). Post-hoc analysis revealed significant increases in levels of ΔFosB and GluR2 expression in the CO group compared with the NC group (negative controls) (ΔFosB: *p* < 0.05; GluR2: *p* < 0.01). ΔFosB and GluR2 levels were significantly reduced in the EA group compared with the CO group (both *p* < 0.05). Western blot data also revealed that the CO + EA + GBP group and CO group had significantly higher levels of ΔFosB (*p* < 0.05) and GluR2 expression (*p* < 0.001) compared with mice given EA only (CO + EA group), suggesting that the combination of GBP and EA significantly reversed the effects of EA (Fig. 3A–C). To test the effects of only EA (at LI4 and LI11) and only GBP treatment in the controls, ΔFosB and GluR2 levels were compared in three groups (see Fig. 4A for the timeline). The results revealed that ΔFosB and GluR2 levels were not changed by EA in controls (Fig. 4B and Supplementary Fig. S5) and by GBP treatment in controls (Fig. 4C and Supplementary Fig. S6).

### EA pretreatment at LI4 and LI11 prevented increases in dendritic spine density induced by 2 weeks of cocaine administration

**Figure 5:**
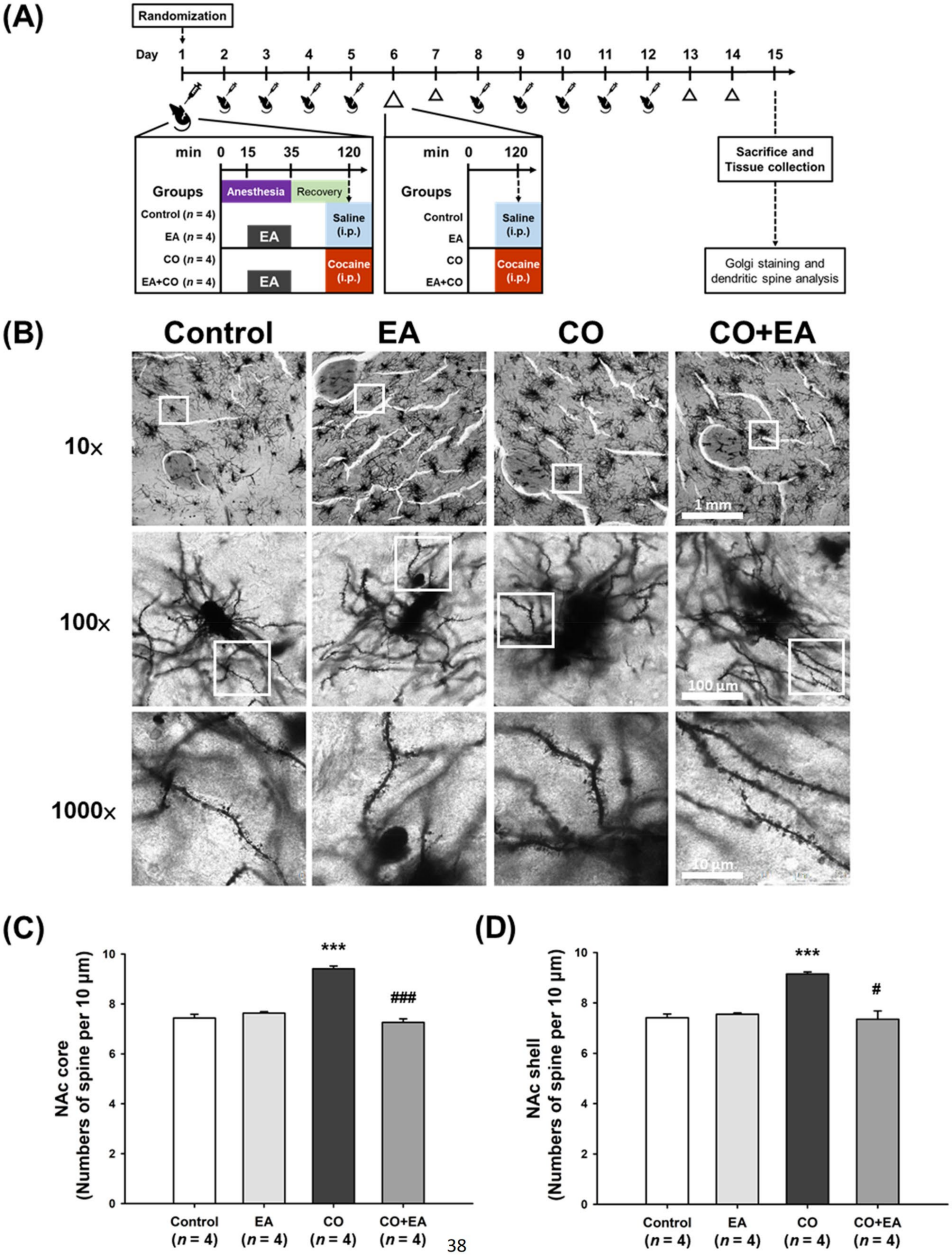
EA pretreatment prevented increases in dendritic spine density induced by 2 weeks of cocaine administration. (**A**) Timeline for the experimental procedure. (**B**) On Day 15, the mice were sacrificed for Golgi-Cox staining. Representative laser confocal photomicrographs of dendritic processes were obtained from the NAc of all mice in each group. The white boxes indicate the areas of interest examined under 100× and 1000× magnification conditions, respectively. (**C**, **D**) Bar graphs showing levels of spine density in the NAc core and shell. Statistical testing was performed by two-way ANOVA with post-hoc *t*-testing. All groups were anesthetized and maintained under 1.5% isoflurane inhalation. All data are expressed as the means ± S.E.M. (*n* = 4 mice per group, 20 fragments each measuring 10 µm obtained from 3 to 5 neurons per sample). ***p < 0.001 versus Control group, ^#^*p* < 0.05, ^###^*p* < 0.001 versus CO group. Abbreviations: Control = Saline only; EA = EA and saline treatment; CO = cocaine treatment only; CO + EA = EA treatment followed by i.p. cocaine (30 mg/kg).

Dendritic spine density in the NAc core and shell was markedly increased by cocaine (30 mg/kg per day, i.p.) given for 5 consecutive days followed by 2 injection-free days for 4 weeks in total^41^, while a lower dosage (15 mg/kg per day for 8 consecutive days) increased spine density only in the shell^42^ and in another study, cocaine (15 mg/kg per day for 14 consecutive days) induced significant increases in dendritic spine density in the NAc core but had only minimal effects in the NAc shell^43^. Based on the results of these studies, we decided to test the effects of cocaine at the dose of 30 mg/kg (or saline) daily for 14 days (see Fig. 5A for the timeline). Two-way AVOVA analysis indicated significant interaction effects between cocaine administration (including saline and cocaine conditions) and EA treatment (including anesthesia-only and EA conditions) were observed in dendritic spine density in both the NAc core (*F*[1,12] = 97.099, *p* < 0.001) and shell (*F*[1,12] = 26.944, *p* < 0.001) regions (Fig. 5C, D; Supplementary Figs. S7 and S8). Compared with the control group (saline with anesthesia only), neuronal dendritic spine density was significantly increased in both the NAc core (*p* < 0.001) and shell (*p* < 0.001) regions of the CO group (cocaine with anesthesia only), but not in EA-treated mice (saline with anesthesia and EA [EA group]) (Fig. 5C, D). EA pretreatment at LI4 and LI11 in cocaine-treated mice (CO + EA group) was associated with significant restorations in dendritic spine density in both the NAc core (*p* < 0.001) and shell (*p* < 0.05) regions compared with the CO group. Dendritic spine density in the NAc core and shell regions did not differ between the EA group and CO + EA group (Fig. 5C, D).

## Discussion

Scant information is available as to the mechanisms underlying the effects of acupuncture in the treatment of cocaine reinstatement. This study investigated the effects of EA treatment in a murine model of cocaine reinstatement by assessing changes in behavior and specific biomarkers that predict the risk of cocaine reinstatement.

LI4 is an acupoint that is frequently used for treatment of drug addiction^17,44,45^. LI11, another acupoint on the radial nerve, is commonly combined with LI4 in clinical trials^46,47^. We therefore selected the combination of LI4 and LI11 for this EA treatment investigation; both acupoints are positioned near the radial nerve in humans and mice. Previous studies have indicated that different frequencies of EA may have different effects on the release of neuropeptides and neurotransmitters^48^. For instance, EA 2 Hz stimulates the release of endomorphin, enkephalin and endorphin, while EA 100 Hz stimulates the release of dynorphin^49^. EA at 2 Hz and 2/100 Hz, but not at 100 Hz, has been shown to suppress morphine-induced CPP in rats^50^, while other research has shown that 100 Hz EA attenuates morphine-induced CPP and that this effect is completely blocked by δ- and κ-opioid receptor antagonists, which suggests that the activation of δ- and κ-opioid receptors mediates the anticraving effects induced by 100 Hz EA^51^.

EA at 2 Hz has been shown to increase preproenkephalin mRNA levels, whereas EA 100 Hz increases preprodynorphin mRNA levels in the NAc^52^. It is therefore assumed that the inhibitory effects of 2 Hz EA on morphine-induced CPP expression are mediated by the μ- and δ-opioid receptors in the NAc^53^. As to cocaine addiction in basic research, EA 100 Hz has been shown to suppress cocaine-induced CPP^54^, while more recent studies have suggested that EA 2 Hz at acupoint HT7 may suppress cocaine-seeking behavior in rats^20^, as well as methamphetamine-induced affective states and locomotor activity^55^. Thus, the evidence suggests that EA at 2 Hz is effective for inhibiting both morphine- and cocaine-induced addiction. In the present study, we selected EA 2 Hz to examine the effects of EA on cocaine-induced reinstatement. We found that EA 2 Hz at LI4 and LI11 successfully inhibited cocaine-induced CPP reinstatement, suggesting that this EA treatment protocol is appropriate for preventing the reinstatement of cocaine-seeking behavior.

In experimental studies, acupuncture with manual twisting for 1 min^56^ or acupuncture for 20 s^57^ at HT7, but not at LI5, reduced foot shock-induced reinstatement cocaine-seeking behavior^56^ or cocaine-induced locomotion activity^57^. Similarly, acupuncture at HT7, but not at LI5, markedly reduced reinstatement of cocaine-seeking, c-Fos expression and pCREB activation in the NAc shell^56^, and reduced self-administration behavior^58^. LI4 and LI5 are located on the same meridian and near the radial nerve. In the present study, we compared the effects of EA and manual acupuncture without twisting at LI4 and LI11, for longer periods of time than those recorded in previous studies. We found that simple needling at LI4 and LI11 did not produce any significant effects on cocaine reinstatement, consistent with previous findings, while electrical stimulation at LI4 and LI11 prevented the cocaine reinstatement of CPP.

The structure of GBP is similar to that of GABA^59^. However, GBP is not active at GABA receptors and does not modulate GABA transport or metabolism^60,61^. Inhibition of α_2_δ-containing voltage-dependent calcium channels (VDCCs) by GBP appears to be responsible for its anticonvulsant, analgesic and anxiolytic effects^60,62^. A previous study has reported that the combination of GBP (3 mg/kg) and EA at ST36 was associated with more effective analgesia than the use of either treatment alone and that the mechanism underlying this synergistic action involved the interaction of opioidergic, noradrenergic and cholinergic systems in the central nervous system^35^. In our investigation, our results (Figs. 2, 3) unexpectedly showed that GBP 1 mg/kg alone (CO + GBP group) or in combination with EA (CO + EA + GBP group) failed to protect mice from cocaine relapse. The addition of GBP to EA reversed the effects of EA at LI4 and LI11 upon cocaine-induced increases in levels of ΔFosB and GluR2. GBP (1,200–3,200 mg/day) effectively treats alcohol withdrawal syndrome by reducing craving, improving rates of abstinence and reducing the likelihood of heavy drinking^63^. GBP may have some therapeutic potential in the treatment of opioid addiction and cannabis dependence, but there is no significant evidence in support of its use for cocaine and amphetamine abuse^63^. One review has noted that GBP has the potential for misuse when patients consume a larger dose than has been prescribed or they use GBP without prescription^64^. Patients have stated that they use GBP to enhance pain relief and also to help with withdrawal symptoms from overuse of substances such as cocaine, buprenorphine and oxycodone, citing its advantages as being inexpensive and “always available”^65^. Such findings suggest that if GBP is prescribed to treat drug addiction, patients could misuse the drug in combination with opioids and stimulants and thereby increase the risk of abuse, rather than enhance the rates of successful rehabilitation. Furthermore, our study suggests that if the patient receives EA for detoxification from illicit substances or alcohol addiction and is prescribed concomitant GBP, the effects of EA might be negated by GBP. Further preclinical research is needed to clarify the molecular mechanisms underlying the effects of GBP in combination with EA, such as whether the inhibition of VDCCs might reverse the effects of EA when used to treat psychostimulant addiction, and to determine which brain area is affected most in addiction by GBP and its involvement in the addiction cycle.

ΔFosB is induced in D1-type NAc medium spiny neurons in response to chronic consumption of various natural rewards, such as high-fat food, sucrose, sex, and wheel running^13^. A similar pathological addictive state is seen with ΔFosB overexpression in drug addiction^66^. ΔFosB overexpression in D1-type medium spiny neurons in the NAc is essential for many of the neural adaptations and behavioral effects seen in animal models of drug addiction^67^.

Cocaine sensitization and chronic stress studies have confirmed correlations in the overexpression of ΔFosB and one of its target genes, GluR2^11^. Other researchers have observed an increase in AMPA receptor function after psychostimulant exposure^68^, which is supported by research showing that repeated exposure to cocaine followed by a period of withdrawal increases levels of synaptic AMPA receptors in the NAc region^69^. In another rat study, 7 days of cocaine administration followed by 14 days of withdrawal and then a challenge dose of cocaine increased the surface expression of AMPA GluR1 and GluR2 in the NAc^70^. In our study, cocaine-induced increases in AMPA GluR2 and ΔFosB levels were observed after 10 days of withdrawal, while these effects were reduced by treatment with EA at LI4 and LI11. In the present study, GBP not only reversed the effects of EA on CPP reinstatement, but also reversed EA-induced reductions in ΔFosB and GluR2 levels. These results indicate that ΔFosB and GluR2 levels may be closely associated with CPP reinstatement. However, detailed interactions between cocaine, EA and GBP at the protein levels of ΔFosB and GluR2 have not been fully explored in the present study. A 2 × 2 × 2 design (3-way ANOVA: drug x pharmacological treatment × EA) may provide full information and could be warranted for future study.

Chronic administration of cocaine and also methamphetamine increases dendritic branching and density in the prefrontal cortex and the NAc^12^. In experiments using transgenic mice that overexpress ΔFosB, chronic cocaine administration increased the number of ΔFosB-regulated glutamatergic synapses in the NAc, while other studies have reported that both cocaine and methylphenidate increase ΔFosB expression and also dendritic spine density on medium spiny neurons^9,71^. Changes in the size and shape of individual dendritic spines correlate with long-term potentiation and long-term depression^72,73^. The induction of long-term potentiation is associated with the formation of new spines^74^. These alterations might reflect stable changes of neurons that are associated with the long-term behavioral changes seen with addiction^75^.

**Figure 6:**
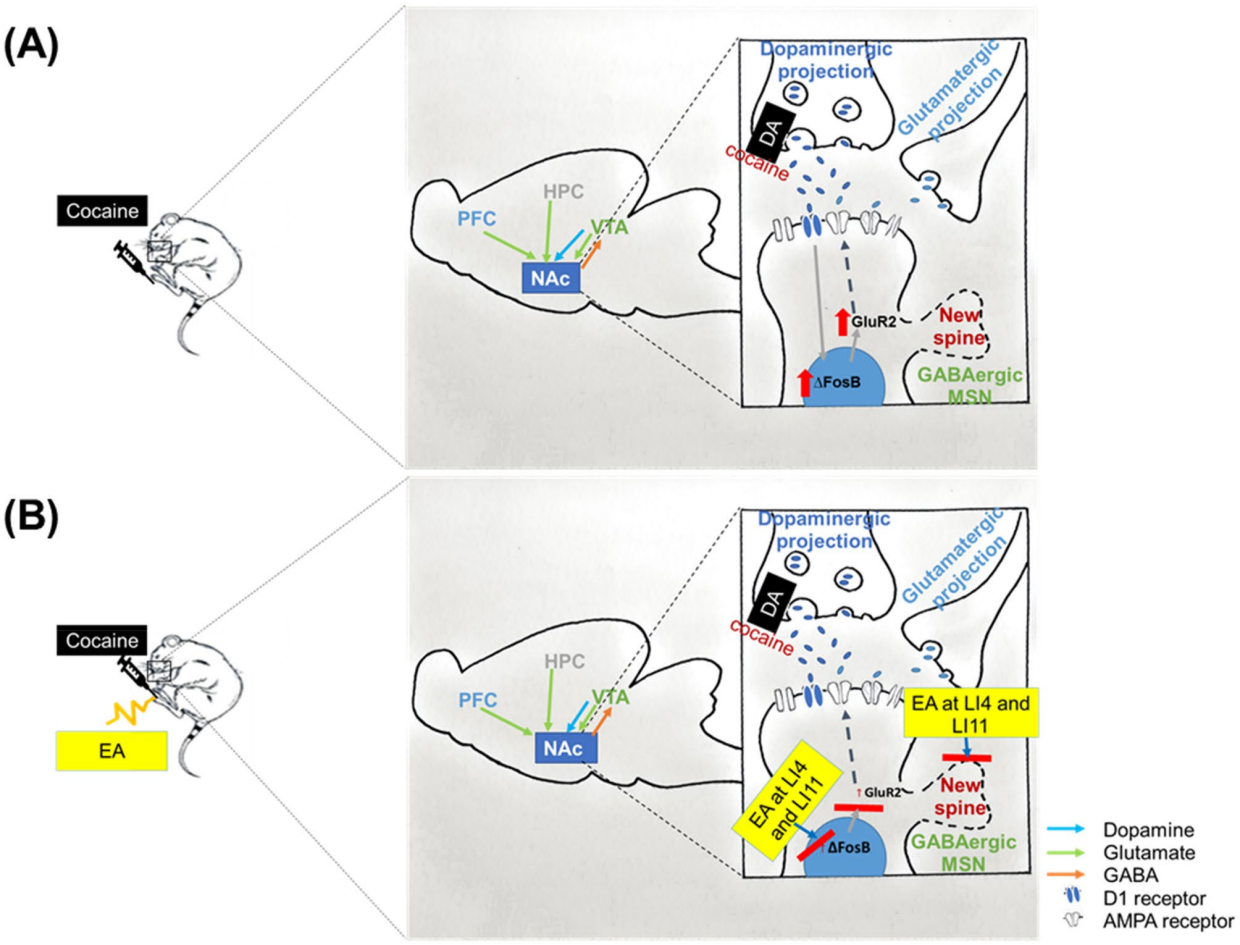
Schematic diagram showing synaptic events that occur after exposure to cocaine and subsequent events occurring in the NAc. (**A**) Cocaine upregulates levels of ΔFosB and GluR2 expression in the NAc. Chronic cocaine increases dendritic spine density in the NAc. (**B**) EA at LI4 and L11 reverses cocaine-induced changes in levels of ΔFosB and GluR2 expression in the NAc and also reverses the increases in dendritic spine density.

Our study followed a previously used method of repeated cocaine administration in rodents to induce increases in dendritic spine density on medium spiny neurons in the NAc^49^. In our study, after 2 weeks of cocaine administration, dendritic spine density was significantly increased in both the NAc core and shell regions. These increases were prevented to a significant extent by EA pretreatment at LI4 and LI11, apparently by maintaining dendritic spine density despite chronic cocaine administration. In conclusion, these findings suggest that EA at LI4 and LI11 may prevent cocaine-induced reinstatement behavior, by modulating ΔFosB and GluR2 levels, as well as reducing dendritic spine density. A schematic diagram (Fig. 6) shows synaptic events that occur after exposure to cocaine and subsequent events occurring in the NAc.

Golgi staining data have revealed that repeated cocaine injections stimulate spinogenesis of NAc medium spiny neurons^75^. We used the same methodology in this study to analyze the effects of cocaine upon dendritic morphology, as Golgi staining is a commonly used method to visualize changes in neuronal morphology without requiring specific protein labeling. In consideration of doses and durations of cocaine regimens used in previous studies^41,43^, our study administered cocaine 30 mg/kg for 2 weeks to induce increases in dendritic spine density in both the NAc core and shell. By combining more advanced methods (the DiOlistic technique, immunostaining, three-dimensional morphometric analysis and time-lapse two-photon microscopy), a recent study demonstrated that acute exposure to cocaine (10 mg/kg and 20 mg/kg, i.p.) triggered rapid synaptogenesis and a persistent increase in glutamatergic connectivity in striatal projection neurons from the NAc shell that subsequently altered behavior in alive mice^76^. Based on the findings of a previous study, in which ΔFosB increased the density of immature dendritic spines in D1 dopamine receptor-expressing direct pathway medium spiny neurons in both the NAc shell and core^9^, we assumed that increased NAc spine density is linked to ΔFosB expression. However, it is difficult to use an ordinary Golgi staining method to evaluate the precise molecular processes involved in spinogenesis after lower dose, shorter durations of cocaine regimens, as discussed by other researchers^76^.

Although we now assume that the 4 doses of cocaine used in our CPP paradigm can cause spinogenesis and are link to the observed changes in behavior and ΔFosB expression, future work will be needed to demonstrate evidence in support of this assumption. Such research should employ more advanced techniques such as three-dimensional morphometric analysis and time-lapse two-photon microscopy to simultaneously quantify any correlation between cocaine-induced spinogenesis and ΔFosB overexpression with behavioral alterations, and to determine whether EA has any effects on such correlations.

## Materials and methods

### Experimental animals and ethical considerations

Male ICR mice (4–6 weeks, weighing 25–30 g) were obtained from BioLASCO Taiwan Co., Ltd (Taiwan) and acclimatized to the environment, then maintained at 25 °C under a 12-h light–dark cycle for at least 1 week before the experiments were performed. They had free access to pellets and drinking water. Mice were bred in a similar environment to avoid unintended consequences of environmental enrichment. All experimental procedures were conducted between 08:00 and 18:00 h. On each day of behavioral testing, the order of testing was by random assignment. The injection procedures were carried out in the home cage. All experiments in this study were approved by the China Medical University Institutional Animal Care and Use Committee (approval number: CMUIACUC-2019–077) and were performed in accordance with the Chinese Taipei Society of Laboratory Animal Sciences guidelines and ARRIVE (Animal Research: Reporting of In Vivo Experiments) guidelines (https://arriveguidelines.org/ arrive-guidelines).

### Study drug

Cocaine was acquired from the Factory for Controlled Drugs, Food and Drug Administration, Ministry of Health and Welfare (Taipei, Taiwan). GBP was purchased from ACROS Organics (Fisher Scientific Inc., Pittsburgh, PA, USA) and lidocaine was purchased from Sigma‐Aldrich (St Louis, MO, USA). Drugs were dissolved in sterile saline (distilled 0.9% sodium chloride) then administered by i.p. or local injections.

### Conditioned place preference (CPP) model

Before entering the experimental phase, mice were placed in the animal behavior room for 1 h of acclimation. CPP was conducted using a 2-chambered box (black and white), with each chamber measuring the same size (15 × 15 × 15 cm^3^). As per previous research^39,43,77^, the CPP experiment was designed to mimic 4 testing stages of cocaine seeking in mice (preconditioning [PRE], post-conditioning [POST], extinction [EXT], and reinstatement [REIN]).

### Preconditioning (PRE) testing

The conditioning compartments were separated by a guillotine trap door, which was open on Day 1. Each mouse was randomly placed into one of the compartments and allowed to move freely between them for 20 min to determine PRE place preference. Mice were excluded from the study if they spent more time in the non-preferred (white, cocaine-paired) chamber and if the difference spent in either chamber was < 100 s.

### Post-conditioning (POST) testing

After a single cocaine hydrochloride 20 mg/kg injection in the morning (from 8:00 AM), the mouse was placed in the non-preferred white (cocaine-paired) chamber for 30 min. For the 30-min training sessions, the guillotine trap door was closed. After a single saline injection in the afternoon (from 4:00 PM), the mouse was placed in the preferred black (saline-paired) chamber. This procedure was modified from a previous study^39^ and repeated for 3 consecutive days (Days 2, 3 and 4). On Day 5, the mice were allowed to freely access the two chambers and the time spent in individual chambers was monitored for 20 min. The CPP score was calculated as the time spent in the cocaine-paired end chamber^36,78^.

### Extinction of cocaine CPP (EXT) testing

The mice were trained on two days (Days 14 and 15) without cocaine injections and were randomly placed in either chamber, then tested for preference on Day 16 (EXT testing).

### Cocaine-primed reinstatement (REIN) testing

One day after EXT, the mice underwent acute cocaine challenge with a priming dose of i.p. cocaine (10 mg/kg) followed by immediate REIN testing^36,78^.

### Electroacupuncture and manual acupuncture

All mice were anesthetized and maintained under 1.5% isoflurane inhalation (Panion & BF Biotech Inc., Taoyuan, Taiwan) for acupuncture treatment. EA treatment was applied to the LI4 (Hegu) and LI11 (Quchi) acupoints, which are positioned near the radial nerve on the left forefoot. LI4 is located on the first dorsal interossei, medial to the middle of the second metacarpal bone. The LI11 is located at the depression medial to the extensor carpi radialis, at the lateral end of the cubital crease^79,80^. Stainless steel acupuncture needles (Shanghai Yanglong Medical Articles Co., Ltd., Shanghai, China) measuring 0.2 mm in diameter and 2 cm in length were used in the study. Electrical stimulation (150-µs pulses at 2 Hz for 20 min) was applied via two needles inserted to a depth of 2–3 mm (Supplementary Fig. S9) using an electrical stimulator (Trio 300, Ito, Japan), as per the methodology described in a previous study^79^. Manual acupuncture (MA) was applied to the same acupoints (LI4 and LI11) without electrical stimulation.

### Drug treatment

The cocaine-induced CPP condition involved daily i.p. cocaine (20 mg/kg/day) injections for 3 days (Days 2–4) after the mice completed PRE testing. They received a priming dose i.p. cocaine injection (10 mg/kg) on Day 17 before REIN testing. To examine the effect of radial nerve block on EA treatment, a single local injection of lidocaine (2%, 10 μL) was administered near the LI11 acupoint (proximal 2.0 mm)^81^ 1 min prior to EA treatment. To examine the effect of GBP in combination with EA, GBP (1 mg/kg, i.p.) was injected 2 h after EA treatment.

### CPP experimental groups

The initial investigation into the effects of EA on cocaine-induced CPP reinstatement after PRE testing enrolled 28 mice (aged 5–6 weeks) and treated them with cocaine (20 mg/kg, i.p.) for 3 days (Days 2–4) (Fig. 1A). After 2 h of POST testing on Day 5, the mice were randomly assigned to different experimental conditions: no intervention (CO group); EA treatment (CO + EA group); MA treatment (CO + MA group); or lidocaine (2%, 10 μL) followed by EA treatment (CO + LIDO + EA group), for 8 days.

Subsequent CPP testing examined the effects of GBP alone and GBP combined with EA on cocaine-induced CPP reinstatement (Fig. 2A, B). Twenty-nine mice pretreated with cocaine (20 mg/kg, i.p.) were randomly assigned to 1 of 4 groups after POST testing: no intervention (CO group); EA treatment (CO + EA group); i.p. GBP (1 mg/kg) injection (CO + GBP group); or EA followed by i.p. GBP (1 mg/kg) injection (CO + EA + GBP group), for 8 days. During the study treatment period (Days 6–13), all interventions were performed once daily. The mice were then subjected to extinction training (Days 14 and 15), before EXT testing (Day 16) and REIN testing (Day 17).

### Western blot

Two h after the final CPP testing, the animals were deeply anesthetized before decapitation and then NAc tissue was quickly collected and frozen at − 80 °C. Tissue samples were homogenized in solution containing lysis buffer, protease inhibitors and phosphatase inhibitors (100:1:1). Each sample of protein concentration was evaluated using a Pierce BCA Protein Assay Kit. Proteins (30 μg) were resolved using 8–12% sodium dodecyl sulfate–polyacrylamide gel electrophoresis (SDS-PAGE) under reducing conditions and the gel was transferred to polyvinylidene difluoride (PVDF) membranes, then blocked in 2% BSA (Sigma-Aldrich). In order to simultaneously detect levels of AMPA GluR2 and ΔFosB expression on a single membrane, we cut each membrane according to molecular weight prior to hybridization with primary antibodies. The membranes were incubated overnight at 4 °C with AMPA receptor 2 (GluR2) (1:1,000; #13607S; Cell Signaling), ΔFosB (1:1,000; 14,695; Cell Signaling), or β-actin antibody (1:10,000; GTX629630; GeneTex). Membranes were incubated with secondary antibody (1:10,000; anti-rabbit IgG-HRP sc-2004; anti-mouse IgG-HRP sc-2005). All antibodies were diluted in Tris-buffered saline (TBS) buffer for use. Protein bands were detected and pixel intensity was determined by UN-SCAN-IT gel 6.1 software (Silk Scientific, Inc., Orem, UT, USA) then normalized to β-actin levels.

### Golgi staining and dendritic spine analysis

Sixteen male ICR mice (aged 4 weeks) were randomly grouped into 4 groups: i.p. injection of saline (Control group); i.p. injection of saline after EA (EA group); i.p. injection of cocaine (CO group); i.p. injection of cocaine after EA (CO + EA group). The experiment was designed as a two-stage procedure (see Fig. 5A for the timeline): the first stage involved Days 1–5 and the second involved Days 8–12. On each experimental day, mice were anesthetized for 15 min before being subjected to 20 min of anesthesia with EA or no EA. Two h after treatment, mice received i.p. injections with saline or cocaine (30 mg/kg)^42^. The mice received i.p. injections for 14 consecutive days (including Days 6–7 and 13–14), as per previous research^43^. The mice were sacrificed and their brains collected for Golgi-Cox staining on Day 15. The brains were removed and analyzed by the FD Rapid GolgiStain Kit, as per the manufacturer’s instructions. 125-µm-thick sections were cut using a vibratome, as previously described^82^. Dendritic images of medium spiny neurons were acquired at high resolution to ensure sufficient resolution to conduct spine counting. Photomicrographs were obtained with a confocal laser scanning microscope (Leica TCS SP8 X) with an oil-immersion lens. All measurements were made manually and quantified as previously described^12,83^. Dendritic spines were counted along dendritic processes extending from the soma of fully impregnated medium spiny neurons in both the shell and core of the NAc, as in a previous study^83^. Based on the clarity of the images taken under a total magnification of 1000×, we counted the number of spines/10 µm of 50-µm long dendritic segment (10 in each cerebral hemisphere). Quantitative analysis included only those spines appearing continuous with their parent dendrite shaft in maximum-intensity *z* projection. Mean spine densities were analyzed by pair-wise comparisons using one-way ANOVA with least significant difference (LSD) testing. These measures were obtained from 3–5 neurons in each cerebral hemisphere.

### Statistical analysis

All statistical analyses were performed using SPSS (v20, IBM, Armonk, NY, USA) by a third party who was blinded to the treatment group allocations. Results are expressed as the mean ± standard error of the mean (S.E.M.). For behavior data, the two-way mixed-model ANOVA tested for possible interaction or main effects on group and phase factors. In the event of significant interaction effects, subsequent simple main effects on group and time factors were tested by one-way ANOVA and one-way ANOVA with repeated measurements, respectively. In the event of significant simple main effects, post-hoc analysis was conducted with the LSD test. For Western blot data, one-way ANOVA was conducted to test for possible main effects on between-group factors. In the event of a significant main effect, post-hoc analysis was conducted with the LSD test. For dendritic spine density data, two-way ANOVA tested for possible interactions or main effects after cocaine administration and EA treatment. The level of significance was set at *p* < 0.05.

## Supplementary Information

Supplementary Information.

## Data Availability

All the data generated during this study have been statistically analyzed and are illustrated as figures. The dataset could be available on request from the corresponding author.
