# Supplementary material for: Electroacupuncture prevents cocaine-induced conditioned place preference reinstatement and attenuates ΔFosB and GluR2 expression
Source: Sci Rep. 2021 Jul 1;11:13694. doi: 10.1038/s41598-021-93014-0 (PMC8249658; doi:10.1038/s41598-021-93014-0)
Supplement: Supplementary file 1 — Supplementary Information. [file 41598_2021_93014_MOESM1_ESM.docx]

Supplementary material

for

**Electroacupuncture prevents cocaine-induced conditioned place preference reinstatement and attenuates ΔFosB and GluR2 expression**

Ai T.M. Nguyen, Tran V.B. Quach, Peddanna Kotha, Szu-Yu Chien, Iona J. MacDonald, Hsien-Yuan Lane, Cheng-Hao Tu, Jaung-Geng Lin, and Yi-Hung Chen

**Supplementary Figures S1 – S12**


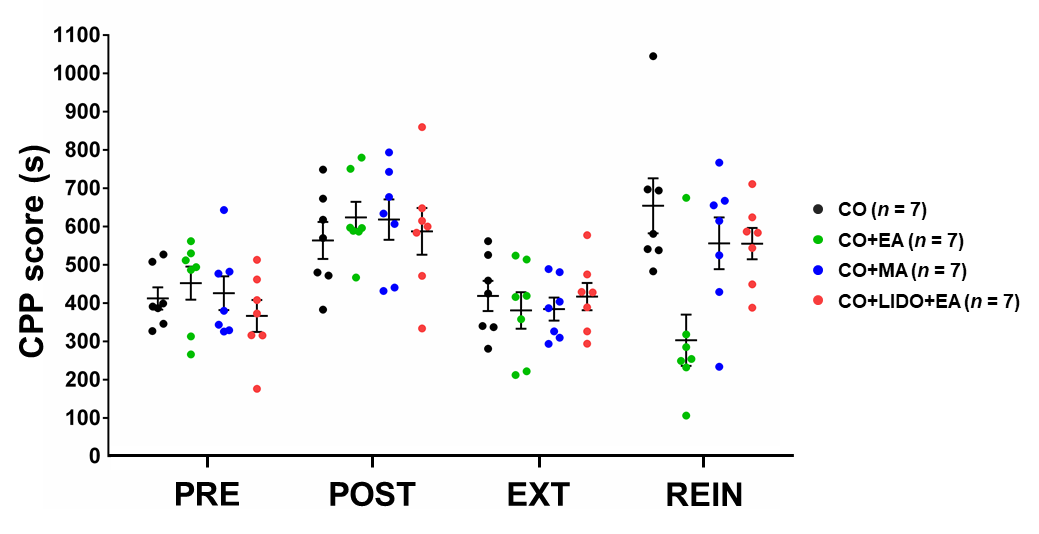


**Supplementary Figure S1.** Individual data points are presented from Figure 1B. Black dot = CO group; green dot = CO+EA group; blue dot = CO+MA group; red dot = CO+LIDO+EA group. Abbreviations: CPP = Cocaine-induced conditioned place preference; PRE = Preconditioning testing; POST = Post-conditioning testing; EXT = Extinction of cocaine CPP testing; REIN = Cocaine-primed reinstatement testing; CO = No intervention; CO+EA = EA treatment; CO+MA = MA treatment; CO+LIDO+EA = Lidocaine (2%, 10 μL) injection followed by EA treatment.


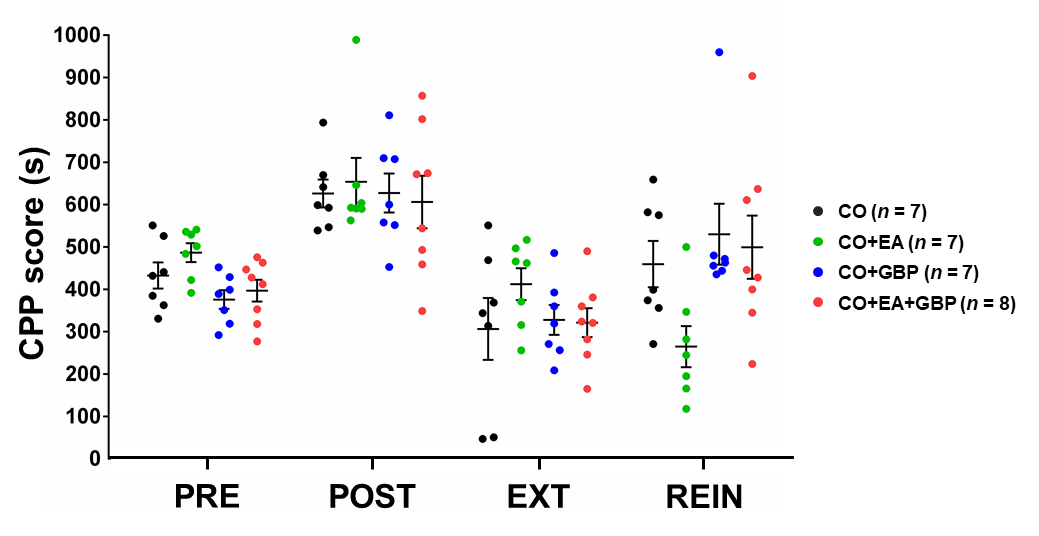


**Supplementary Figure S2.** Individual data points are presented from Figure 2B. Black dot = CO group; green dot = CO+EA group; blue dot = CO+GBP group; red dot = CO+EA+GBP group. Abbreviations: CPP = Cocaine-induced conditioned place preference; PRE = Preconditioning testing; POST = Post-conditioning testing; EXT = Extinction of cocaine CPP testing; REIN = Cocaine-primed reinstatement testing; CO = No intervention; CO+EA = EA treatment; CO+GBP = i.p. GBP (1 mg/kg) injection; CO+EA+GBP = EA treatment followed by i.p. GBP (1 mg/kg) injection.


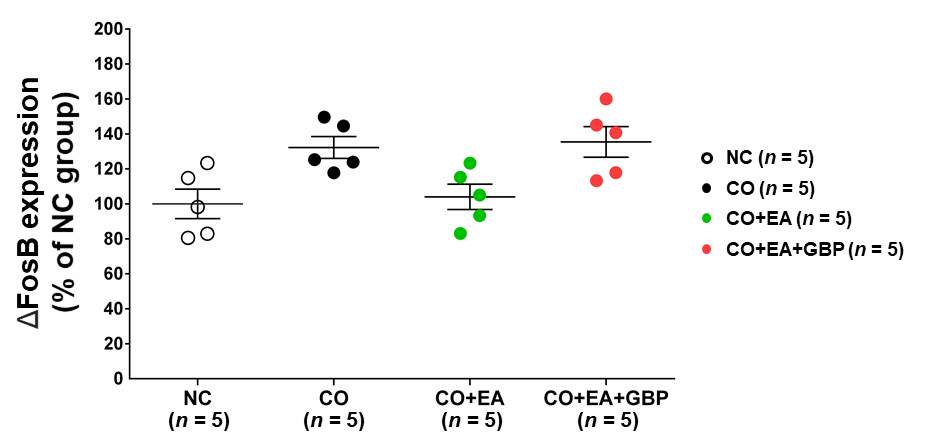


**Supplementary Figure S3.** Individual data points are presented from Figure 3B. Colorless dot = NC group; black dot = CO group; green dot = CO+EA group; red dot = CO+EA+GBP group. Abbreviations: NC = Negative control; CO = No intervention; CO+EA = EA treatment; CO+EA+GBP = EA treatment followed by i.p. GBP (1 mg/kg) injection.


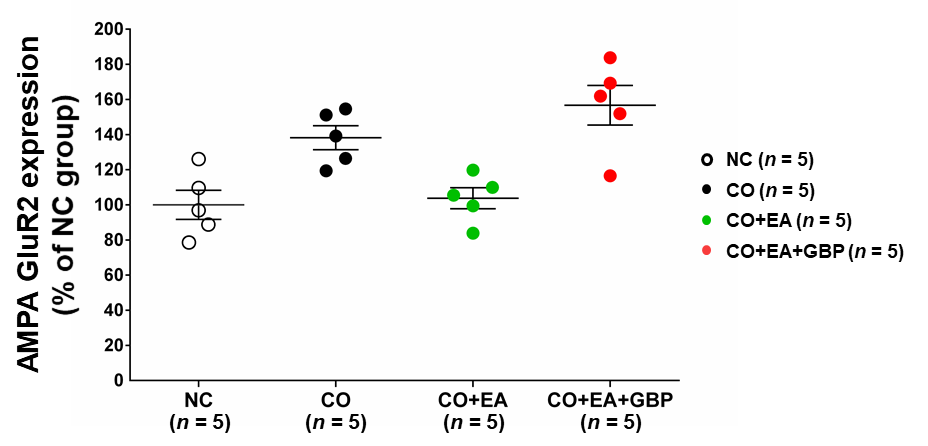


**Supplementary Figure S4.** Individual data points are presented from Figure 3C. Colorless dot = NC group; black dot = CO group; green dot = CO+EA group; red dot = CO+EA+GBP group. Abbreviations: NC = Negative control; CO = No intervention; CO+EA = EA treatment; CO+EA+GBP = EA treatment followed by i.p. GBP (1 mg/kg) injection.


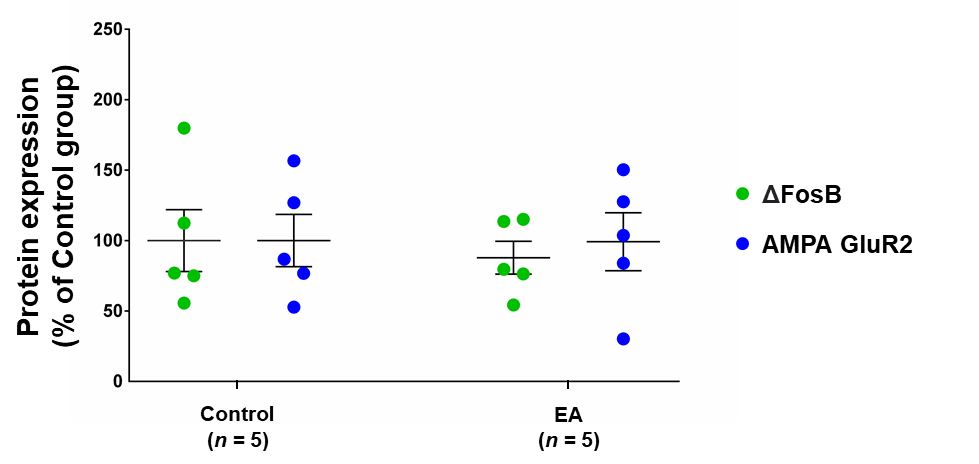


**Supplementary Figure S5.** Individual data points are presented from Figure 4B. Green dot = ΔFosB; blue dot = AMPA GluR2. Abbreviations: Control = Cocaine-naïve mice; EA = cocaine-naïve mice with EA treatment.


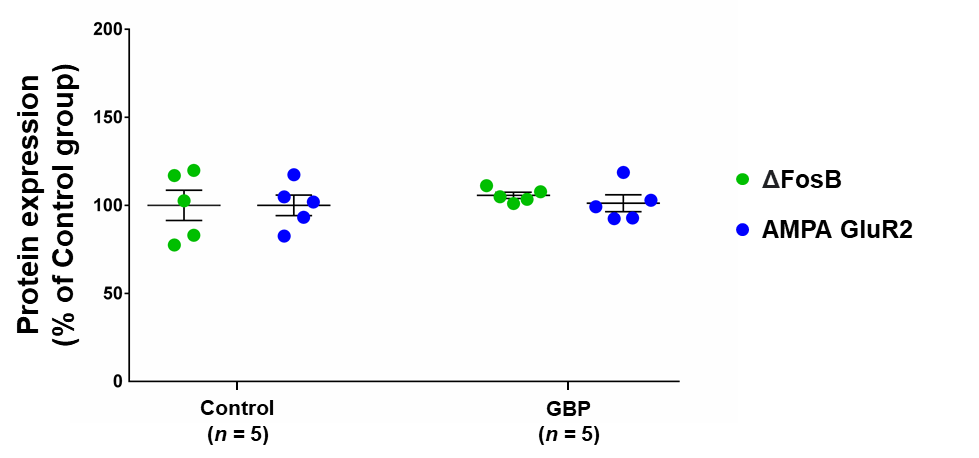


**Supplementary Figure S6.** Individual data points are presented from Figure 4C. Green dot = ΔFosB; blue dot = AMPA GluR2. Abbreviations: Control = Cocaine-naïve mice; GBP = only i.p. GBP (1mg/kg) treatment.


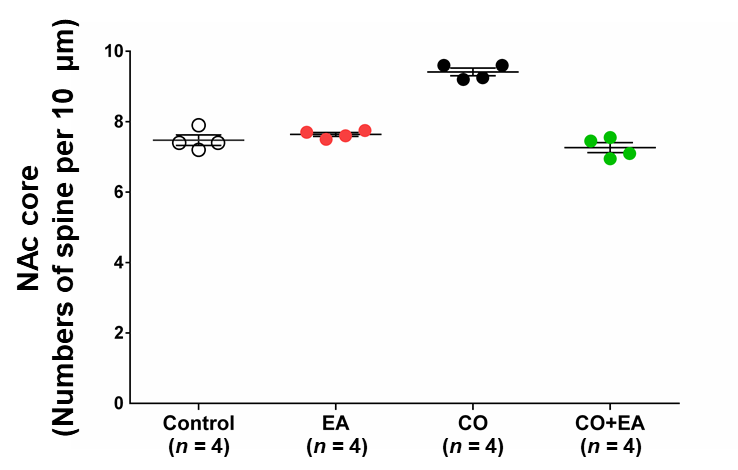


**Supplementary Figure S7.** Individual data points are presented from Figure 5C. Colorless dot = Control group; red dot = EA group; black dot = CO group; green dot = CO+EA group. Abbreviations: Control = Saline only; EA = EA and saline treatment; CO = cocaine treatment only; CO+EA = EA treatment followed by i.p. cocaine (30 mg/kg). All mice were anesthetized and maintained under 1.5% isoflurane inhalation.


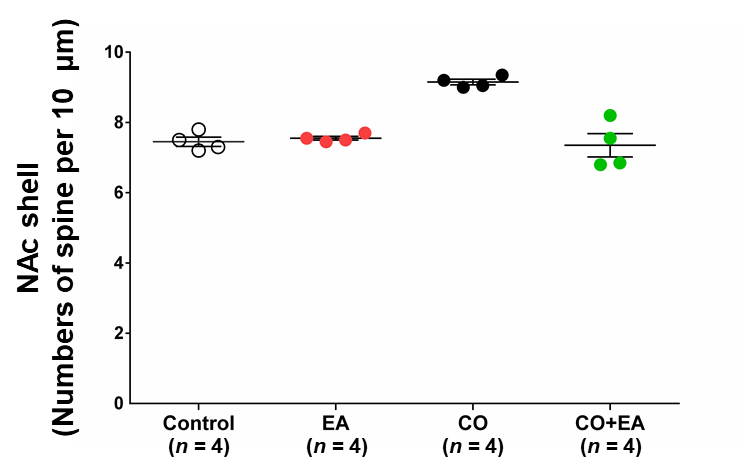


**Supplementary Figure S8.** Individual data points are presented from Figure 5D. Colorless dot = Control group; red dot = EA group; black dot = CO group; green dot = CO+EA group. Abbreviations: Control = Saline only; EA = EA and saline treatment; CO = cocaine treatment only; CO+EA = EA treatment followed by i.p. cocaine (30 mg/kg). All mice were anesthetized and maintained under 1.5% isoflurane inhalation.

| (A) | (B) |
| --- | --- |
| 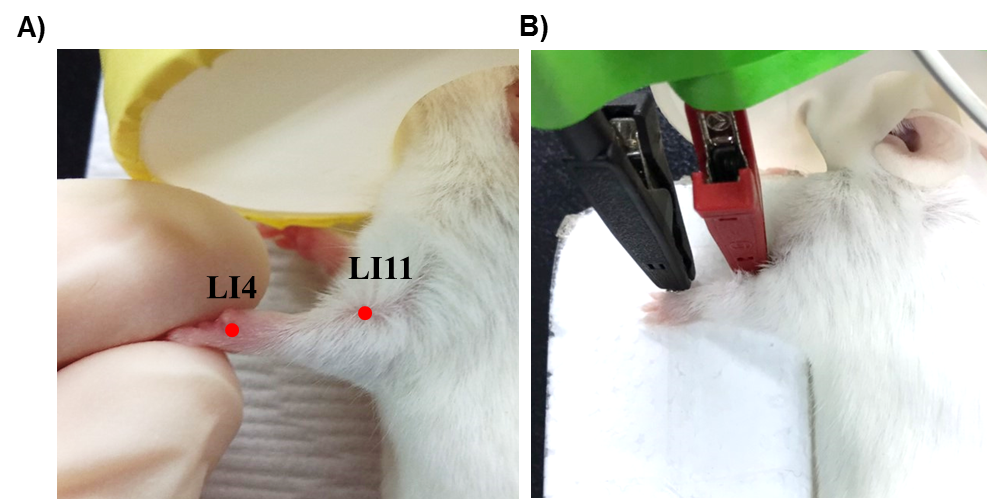 | |

**Supplementary Figure S9.** Illustration of electroacupuncture (EA) treatment. (A) Location of LI4 and LI11 acupoints. (B) Application of EA.

| (A) | |
| --- | --- |
| AMPA GluR2 (100 kDa) | |
| 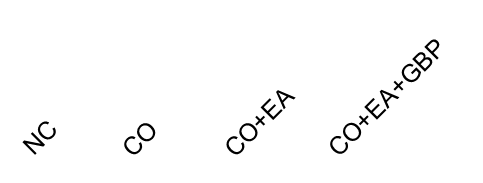 |  |
| ΔFosB (37 kDa) | |
| 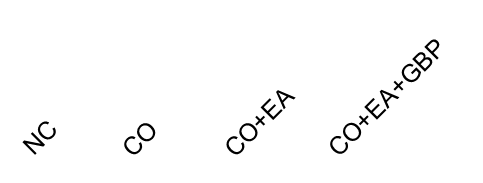 |  |
| β-Actin (42 kDa) | |
| 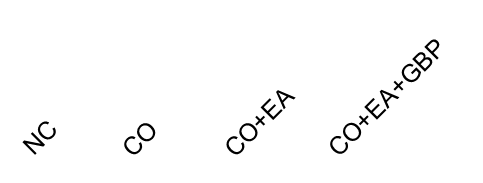 |  |

| (B) Serial exposure times of β-actin blots |  |
| --- | --- |
| 10 seconds | 30 seconds |
|  |  |
| 40 seconds |  |
|  |  |

**Supplementary Figure S10.** Original Western blot images are presented from Figure 3. (A) The selected areas indicated by red dashed lines on the bands (Left) are represented by their respective photographs (Right). (B) β-Actin Western blot images acquired at 10, 30 and 40 seconds, respectively. Abbreviations: NC = Negative control; CO = No intervention; CO+EA = EA treatment; CO+EA+GBP = EA treatment followed by i.p. GBP (1 mg/kg) injection.

| AMPA GluR2 (100 kDa) | |
| --- | --- |
| 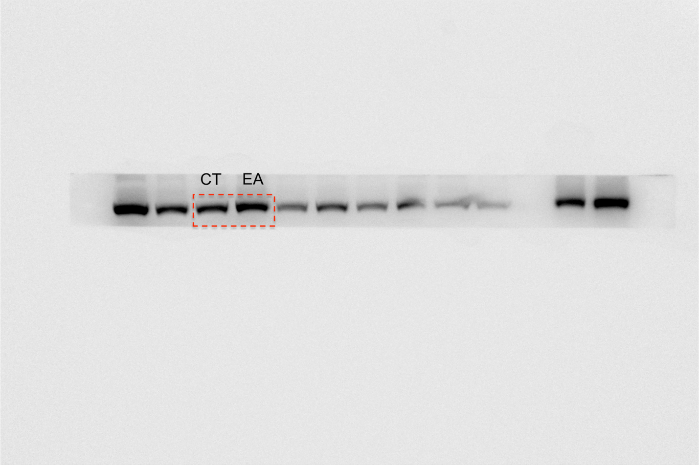 |  |
| ΔFosB (37 kDa) | |
| 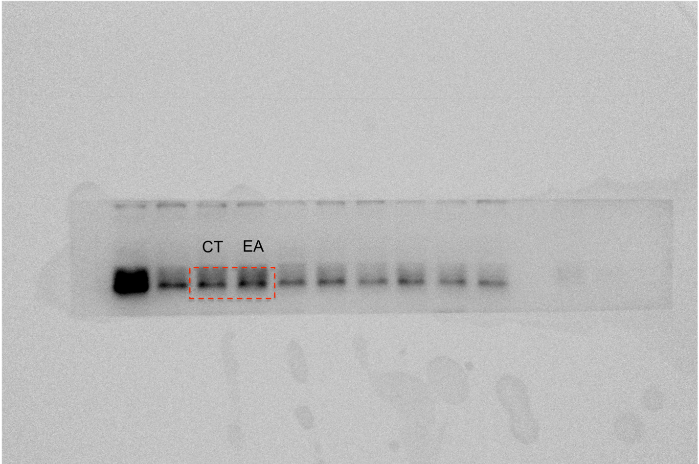 |  |
| β-Actin (42 kDa) | |
| 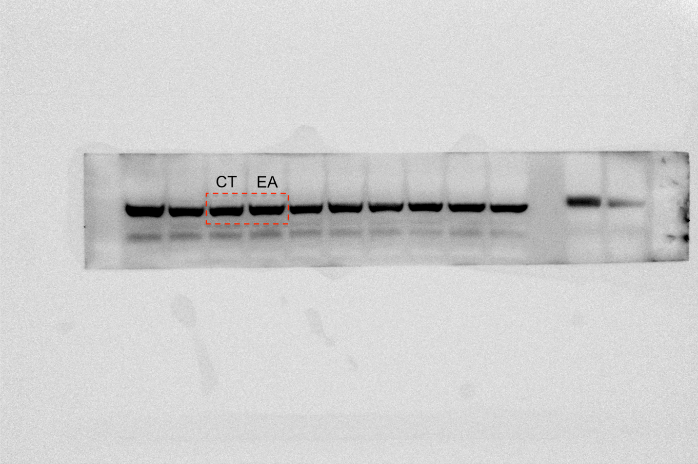 |  |

**Supplementary Figure S11.** Original Western blot images are presented from Figure 4B. The selected areas indicated by red dashed lines on the bands (Left) are represented by their respective photographs (Right). Abbreviations: CT = Control group (Cocaine-naïve mice); EA = EA at LI4 and LI11.

| AMPA GluR2 (100 kDa) | |
| --- | --- |
| 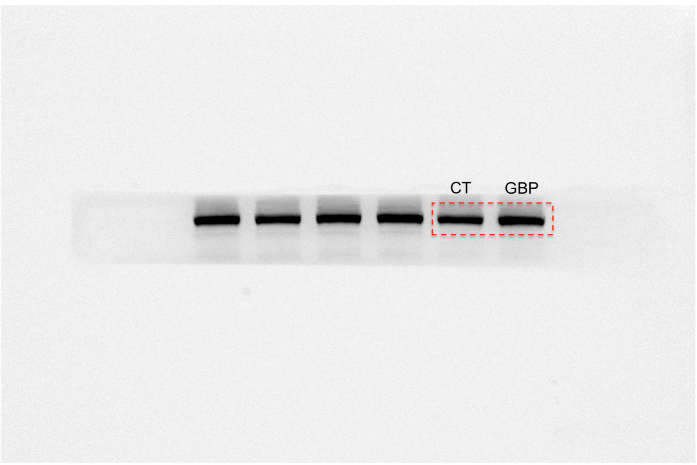 |  |
| ΔFosB (37 kDa) | |
| 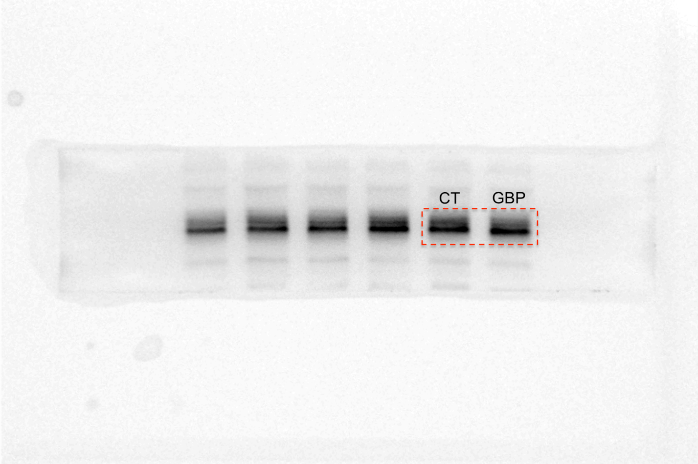 |  |
| β-Actin (42 kDa) | |
| 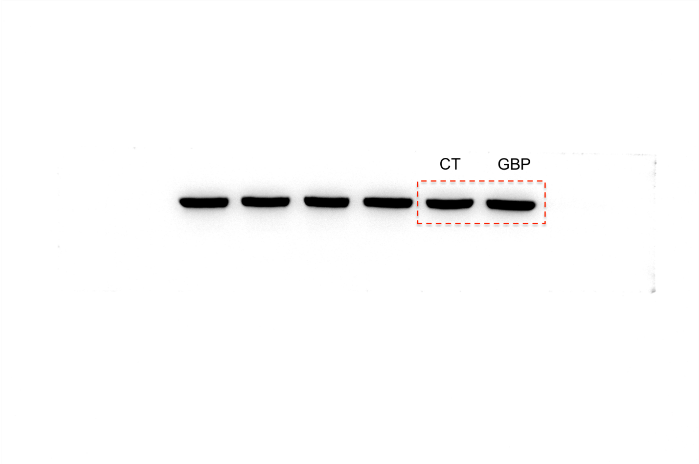 |  |

**Supplementary Figure S12.** Original Western blot images are presented from Figure 4C. The selected areas indicated by red dashed lines on the bands (Left) are represented by their respective photographs (Right). Abbreviations: CT = Control group (Cocaine-naïve mice); GBP = Gabapentin (1 mg/kg, i.p.).
